# Confronting neglected tropical diseases: a moral and strategic imperative for global equity

**DOI:** 10.1186/s40249-026-01414-z

**Published:** 2026-01-29

**Authors:** Dirk Engels, Xiao-Nong Zhou

**Affiliations:** 1 Commugny, Vaud, Switzerland; 2 National Key Laboratory of Intelligent Tracking and Forecasting for Infectious Diseases; National Institute of Parasitic Diseases at Chinese Center for Disease Control and Prevention (Chinese Center for Tropical Diseases Research); Key Laboratory on Parasite and Vector Biology, Ministry of Health; WHO Centre for Tropical Diseases; National Center for International Research on Tropical Diseases, Ministry of Science and Technology, Shanghai, 200025 China; 3 School of Global Health, Chinese Center for Tropical Diseases Research, Shanghai Jiao Tong University School of Medicine, Shanghai, 200025 China; 4 Hainan Center for Tropical Diseases Research (Hainan Sub-Center of Chinese Center for Tropical Diseases Research), Haikou, 571129 China

**Keywords:** Neglected tropical diseases, Equity, Global health security, Universal health coverage, Sustainable Development Goals, Roadmap

## Abstract

This editorial, published on the occasion of the World Neglected Tropical Diseases Day in 2026, highlights the persistent and pressing issue of neglected tropical diseases (NTDs) and their impact on global health equity. It argues that addressing NTDs is not only a moral imperative but also a strategic necessity for achieving global health security and advancing the Sustainable Development Goals (SDGs). The editorial emphasizes the need for integrating NTD control strategies with the SDGs to leverage synergies and maximize impact. For instance, improving water and sanitation (SDG 6) directly contributes to the prevention of schistosomiasis. Additionally, the *Pandemic Agreement*, co-signed by member countries of World Health Organization in 2025, provides an opportunity to mainstream NTDs into health security planning, ensuring resilience against future outbreaks. The editorial concludes by calling for sustained action and increased investment in research and development, One Health approaches, and policy coherence to embed NTDs in pandemic preparedness and universal health coverage agendas. Achieving a future free from NTDs is possible, but it requires unwavering commitment, collaboration, and equitable resource allocation.

## Introduction

Neglected tropical diseases (NTDs) persist as a glaring inequity in global health, afflicting over 1.7 billion people annually in impoverished tropical and subtropical regions. These diseases, encompassing 21 World Health Organization (WHO)-classified infections [1, 2], remain underprioritized despite their devastating health and socioeconomic impacts. This editorial synthesizes the current landscape of NTDs, highlighting their intersection with poverty, their evolving epidemiology, and their critical role in global health security frameworks [3]. As we mark World Neglected Tropical Diseases Day in 2026, renewed commitment is needed to align NTD control with the second WHO Roadmap for NTD (2021–2030) and emergent *Pandemic Agreement*, ensuring these diseases are no longer sidelined in the pursuit of universal health coverage (UHC) and sustainable development [1, 4].

## Defining the challenge

NTDs are a heterogeneous group of infections unified by their predilection for marginalized populations. Their persistence is fueled by poverty-related factors, including inadequate sanitation, limited healthcare access, and environmental conditions that facilitate transmission [5].

Unlike high-mortality epidemics, NTDs often cause chronic disability, such as blindness from trachoma, limb disfigurement from leprosy, or lymphatic filariasis-induced elephantiasis, perpetuating cycles of lost productivity and social exclusion. The WHO list spans protozoan (e.g., Chagas disease), helminthic (e.g., schistosomiasis), bacterial (e.g., Buruli ulcer), viral (e.g., dengue), and ectoparasitic (e.g., scabies) infections. Notably, several NTDs, including dengue and other arbovirus diseases and rabies, exhibit epidemic/pandemic potential, bridging the gap between neglected diseases and global health security agendas [6].

## Epidemiological and socioeconomic burden

Geographically, NTDs cluster in 149 countries, with sub-Saharan Africa, South Asia, and Latin America bearing the highest burdens. Vulnerable groups, including rural populations, urban slum dwellers, and indigenous communities, face compounded risks due to healthcare access barriers. Gender disparities further exacerbate impacts, women and children are disproportionately affected, exemplified by girls contracting schistosomiasis during water-fetching chores. The socioeconomic toll is far-reaching: NTDs cost afflicted households an estimated 20–30% of annual income, trapping generations in poverty. Stigma associated with disfigurement or disability further isolates patients, limiting education and employment opportunities [6].

## Integration into global health frameworks

The *Ending the neglect to attain the Sustainable Development Goals: a road map for neglected tropical diseases 2021–2030*, issued by WHO, marks a paradigm shift, targeting the elimination of at least one NTD in 100 countries and a 90% reduction in disability-adjusted life years (DALYs) [1]. Key strategies include mass drug administration (e.g., for lymphatic filariasis), vector control (e.g., insecticide-treated nets for Chagas disease), and WASH (Water, Sanitation, and Hygiene) interventions. Crucially, the roadmap emphasizes One Health approaches for zoonotic NTDs like rabies, acknowledging the interplay of human, animal, and environmental health [4].

Simultaneously, the *Pandemic Agreement*, issued by WHO at the 2025 World Health Assembly and agreed by all member countries, presents an opportunity to mainstream NTDs into health security planning. Strengthening surveillance for zoonotic NTDs (e.g., leishmaniasis), ensuring equitable access to diagnostics, and safeguarding NTD programmes from resource diversion during pandemics are critical steps. The COVID-19 pandemic underscored how neglecting endemic diseases during crises widens health disparities, it is a lesson that must inform future policies [7].

## Persisting barriers and emerging opportunities

Despite progress in the global NTD programmes, formidable challenges endure. Funding remains precarious, with many programmes reliant on volatile donor support, as the year 2025 has demonstrated. Climate change is expanding vector habitats of *Aedes* mosquitoes which are carriers of dengue and other arboviruses, that now thrive in previously unaffected regions [8]. Drug resistance looms as a threat, particularly for leishmaniasis [9].

Yet, innovative solutions are emerging. Integrating NTD control with the Sustainable Development Goals (SDGs) leverages synergies, for instance, schistosomiasis prevention aligns with SDG 6 (clean water). Digital tools, such as AI-driven disease mapping and mobile health platforms, enhance surveillance in resource-limited settings. Public-private partnerships, exemplified by the important medicine donations that have allowed countries to bring preventive treatment activities to scale, have proven to be a sustainable global intervention resulting in NTD eliminations in 58 countries, that have been able to achieve elimination of 84 NTDs at the national level up to October 2025 [10].

## Conclusion: a call for sustained action

NTDs are not intractable, they are diseases of neglect, amenable to proven interventions. In order to consolidate current achievements and further advance them, the scientific community must advocate for three priorities: (i) increased investment in research and development for novel diagnostics, treatments, and vaccines, coupled with health system strengthening; (ii) One Health integration to address zoonotic spillovers and climate-driven transmission shifts; and (iii) policy coherence to embed NTDs in pandemic preparedness and UHC agendas.

As we commemorate World Neglected Tropical Diseases Day, let us reaffirm that eliminating these diseases is not merely a moral obligation but a strategic imperative for global health security. The tools exist while the missing ingredients are political will and equitable resource allocation. A future free from NTDs is achievable, but only through unwavering, collaborative action.

## Data Availability

Following approval by the National Institute of Parasitic Diseases, Chinese Center for Disease Control and Prevention (Shanghai, China), the datasets underlying the results of this article will be made available to investigators. Please email the corresponding author for more information.
